# Hybrid Cements and Construction Elements Based on Alkaline Activation with Sodium Sulfates from Fly Ash and Construction and Demolition Waste

**DOI:** 10.3390/ma16186272

**Published:** 2023-09-19

**Authors:** William Valencia-Saavedra, Rafael A. Robayo-Salazar, Ruby Mejía de Gutiérrez

**Affiliations:** Composites Materials Group (CENM), School of Materials Engineering, Universidad del Valle, Calle 13 #100-00, E44, Cali 760032, Colombia; william.gustavo.valencia@correounivalle.edu.co (W.V.-S.); rafael.robayo@correounivalle.edu.co (R.A.R.-S.)

**Keywords:** hybrid alkali-activated cements, fly ash, construction and demolition waste, sodium sulfate, concretes, recycled aggregates, precast elements

## Abstract

This article demonstrates the possibility of producing hybrid cementitious materials (pastes, mortars, concretes, and precast elements) based on fly ash (FA) and construction and demolition wastes (CDW) using alkaline activation technology. Sodium sulfate was used as an activator and fine and coarse aggregates were obtained from CDW residues. An addition of Portland cement (OPC) (10 to 30%) allowed for improvement in the mechanical behavior of the hybrid cements and them to be cured at room temperature (25 °C). The FA and CDW cementitious materials obtained compressive strengths of 37 MPa and 32 MPa, respectively. The compressive strength of FA and CDW alkali-activated concretes at 28 days of curing was 22 MPa and 18 MPa, respectively, which identifies them as structural concretes according to NSR-10 title C in Colombia. The potential use of these concretes was validated by obtaining and classifying precast materials.

## 1. Introduction

Climate change is one of the most important environmental problems facing humanity in these times. This is due to the rapid increase in greenhouse gas emissions [1,2]. The construction sector generates the most environmental pollution due to the significant contribution of carbon dioxide (CO_2_) emissions, high energy consumption, and excessive consumption of natural resources. The production of Portland cement is considered a highly intensive process in energy consumption during the different stages of its production. It likewise generates around 900 kg of CO_2_ per ton of cement produced [3,4,5], which represents approximately 5–8% of global human-caused emissions [6,7]. The main CO_2_ emissions in the cement industry come directly from the combustion of fossil fuels (33%) and the calcination of limestone to be converted into calcium oxide; an indirect amount of CO_2_ comes from electricity consumption in the crushing of raw materials and clinker grinding processes (66%) [8]. Currently, reducing CO_2_ generation is the most important environmental objective in the world regarding reducing the atmospheric concentration of greenhouse gases. A document published by UNO Environment (Eco-efficient cements: Potential economically viable solutions for a low-CO_2_ cement-based materials industry) and known as the Cement Technology Road Map published in 2018 shows the technological goals (in energy efficiency, use of alternative fuels, new raw materials, substitution of clinker, carbon capture and storage) that the cement industry must set to reduce CO_2_ emissions by 2050 [9].

These events have led the cement industry and, in general, governments to implement different promising strategies to reduce the accumulation of greenhouse gas emissions in the atmosphere. One of the goals related to the industry indicates that it is necessary to modernize the infrastructure and reconvert industries so that they are sustainable, using resources more efficiently and promoting the adoption of clean and environmentally sound industrial technologies and processes.

One of the alternatives proposed by the scientific community is the use of alkaline activation systems to obtain cementitious materials, which have shown lower energy cost, as well as environmental cost (lower carbon emissions), due to the low temperatures required in their production, generally close to ambient temperature, and to the reuse of industrial byproducts [10,11,12]. These binders result from the chemical interaction between strongly alkaline solutions and a material with high content of amorphous aluminosilicates (fly ash, blast furnace slag, sugarcane bagasse ash, copper slag, construction and demolition waste, among others) [7]. These new materials, in addition to having excellent mechanical performance, even at short ages, present reduced permeability, chemical and thermal stability, thus constituting high performance materials [13]. According to McLellan et al. [14] and Yang et al. [15], alkaline-activated or geopolymer cements can have similar environmental problems during their life cycle to those of Portland cement in terms of CO_2_ emissions and energy requirements; however, according to Tempest et al. [16] most of the emissions from alkaline activation systems can be attributed to the activator solutions due to the energy consumption associated with their production [17]. These controversies arise because in the production of activators such as NaOH and sodium silicate, normally used, high temperature processes are required where carbon emissions are considerably high. Some authors mention that they reach values of 1514–1915 kg/t [18].

Therefore, by using activators with low CO_2_ emissions, the CO_2_ emissions and energy consumption of alkaline activation systems could be greatly reduced. A solid activator, which allows for direct mixing with the precursor, although its contribution to resistance at short ages may be less, is sodium sulfate (Na_2_SO_4_). Unlike sodium silicate and sodium hydroxide, Na_2_SO_4_ can be obtained from natural brines containing sodium sulfate, crystalline evaporation pools, or as a byproduct during the manufacture of various products, such as hydrochloric acid and silica pigments, among others. Therefore, by using sodium sulfate as an activator, a more environmentally friendly cementitious system could be formulated. Also, compared to other alkaline activators, sodium sulfate is usually less expensive and easier to handle due to its lower corrosivity. However, Na_2_SO_4_-based alkaline activation studies are very limited, and have generally been conducted at the level of pastes or mortars. Wang et al. [19] found that the initial compressive strength of mortars alkali activated with Na_2_SO_4_ is lower than that of other activators such as Na_2_CO_3_, NaOH, and sodium silicate; in systems activated with sodium sulfate, compressive strengths of 1.2, 5.1, 10.2, and 20 MPa were observed at ages of 1, 3, 7, and 28 days, respectively. This strength development is lower than that obtained with other activators, which could be related to the low pH (8) presented [2,20,21]. Rashad et al. [22] meanwhile reported that the compressive strength of alkaline activation systems based on slag activated with Na_2_SO_4_ and cured at 40 °C was 30 MPa at 28 days of age, and even the compressive strength could be increased by incorporating silica fume and limestone [23,24]. Velandia et al. [25] evaluated the mechanical behavior of mortars made with 50% different types of fly ash, with variable Fe_2_O_3_ content, and 50% OPC and reported that sodium sulfate does not have the same effect on fly ashes with high Fe_2_O_3_. Hefni et al. [26] studied the influence of different alkaline activators in concrete produced with 40% fly ash and 60% OPC, and although the authors determined that systems activated with sodium sulfate had lower resistance than those activated with sodium silicate at short ages, at long ages the resistances tended to increase and show behaviors similar to those reported by the systems activated with sodium silicate. Villaquirán-Caicedo and Mejía de Gutiérrez [27] activated construction and demolition waste (CDW) using sodium sulfate as an activator, achieving a compressive strength of 18 MPa after 90 days of curing, where they highlight that these results provide an opportunity for the potential reuse of CDW activated with sodium sulfate in new ecological cementitious binders.

Taking into account what has been described above, where it is evident that it is possible to produce alkaline-activated binders based on FA and alkaline-activated CDW using sodium sulfate with satisfactory mechanical properties, in this article, the manufacture of hybrid concrete alkali-activated with sodium sulfate based on FA and CDW was raised. A Portland addition (maximum 30%) and recycled aggregates (fine and coarse), obtained from CDW, were used in the concrete mix. The main engineering properties, such as compressive strength, indirect tensile strength, capillary suction, density, absorption, and porosity, were evaluated. Finally, perforated, solid blocks and paving stones were produced, demonstrating the potential that these concretes have in the manufacture of precast materials. These construction elements meet the specifications and technical standards required for their use and application in the construction sector. It should be noted that it is the first time that results of alkaline activation hybrid concretes based on the mixture of concrete, ceramic, and masonry residues as precursor and aggregates from CDW are reported, using sodium sulfate as activator. As previously was mentioned, sodium sulfate (Na_2_SO_4_), unlike other types of more commercial alkaline activators, is friendlier to the environment, less expensive, and easier to manipulate; it is also solid, which allows for its direct addition to the precursor, allowing for the production of one-part geopolymers.

## 2. Materials and Methods

### 2.1. Characterization of Raw Materials

As raw materials to produce alkali-activated hybrid systems, fly ash (FA), construction and demolition waste (CDW), and ordinary Portland cement (OPC) were used. CDW precursor corresponds to a mixture of concrete (CW), red ceramic (RCW), and masonry wastes (MW), in identical proportions (33.33%)

The chemical composition of these materials, determined with X-ray fluorescence (XRF) using a MagiX-Pro PW-2440 spectrometer (Phillips PANalytical, Tollerton, USA), equipped with a rhodium tube with a maximum power of 4 KW, is included in Table 1. It can be seen in the table that FA is composed of approximately 88.98% of silica, alumina, and iron oxides with an unburned content LOI = 6.35%, so it could be classified as a type F fly ash, as defined in ASTM C618 [28]. CDW meanwhile has relatively high (SiO_2_ + CaO + Al_2_O_3_ = 80.02%) content, and an LOI of 9.86%. Sodium sulfate (Na_2_SO_4_) for industrial use was used as alkaline activator.

**Figure 1:**
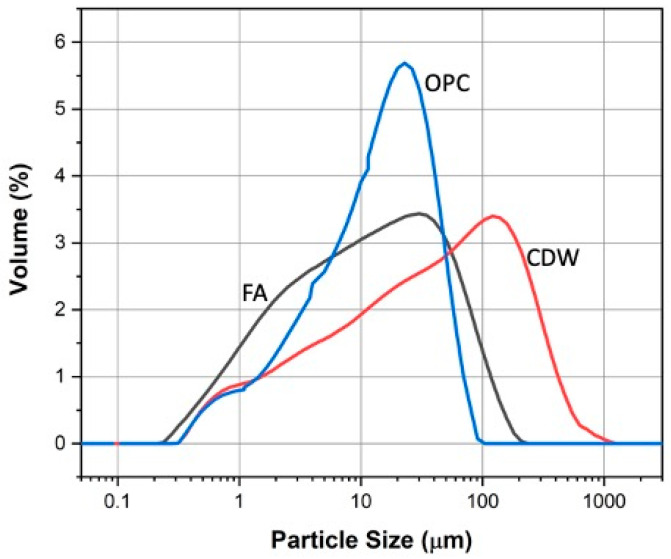
Particle size distribution of the precursors.

The particle size analysis of the FA, CDW, and OPC precursors, carried out by means of laser granulometry in a Mastersizer-2000 (Malvern Instruments equipment, Malvern, UK), resulted in a mean particle size D(4;3) of 36 µm, 75 µm, and 20 µm, respectively. The particle size distribution for these materials is presented in Figure 1. The density of the FA, CDW, and OPC were 2396 kg/m^3^, 2690 kg/m^3^, and 3100 kg/m^3^, respectively.

Table 2 presents the main characteristics of the recycled aggregates obtained from the CDW sample: the coarse aggregate (CRA) from the concrete waste, and the fine aggregate (FRA) from the ceramic and masonry waste. It is noted that these aggregates presented high levels of absorption: FRA 12.12% (ASTM C128, [29]) and CRA 9.17% (ASTM C127, [30]). The high absorption capacity of recycled aggregates is directly related to their nature and high porosity (low density). The physical characteristics obtained allow for its use in mortar and/or concrete mixtures.

### 2.2. Binder Optimization

To optimize the proportions of the Hybrid Cement mixtures (FA/OPC and CDW/OPC), the effect of the Na_2_SO_4_ content in the range of 2–6% and the OPC content (10–30%) in compressive strength at 28 days of curing were evaluated. Once the content of alkaline activator and OPC had been optimized, a comparison was made between the evolution in compressive strength (1–90 days of curing) between the FA/OPC and CDW/OPC (optimal) systems with a reference paste based 100% on OPC (GU type). The hybrid cement pastes were obtained in a Hobart mixer with a mixing time of 5 min; the liquid/solid ratio (L/S) was 0.3 for all systems, which allowed for obtaining good manageability. The pastes were shaped into 20 mm cubes and vibrated for 30 s on an electric vibrating table to remove entrapped air. Subsequently, the molds were covered with a polyethylene film—which allowed for control of the evaporation of free water in the mix—and were cured at room temperature (25 ± 3 °C) at a relative humidity greater than 90% for 24 h, followed by the specimens being removed from the molds and brought to a curing chamber under controlled conditions until the test age. The compressive strength of the pastes was determined in an Instron 3369 universal testing machine (Norwood, MA, USA), which has a capacity of 50 kN force, at a speed of 1 mm/min. For each mix, a minimum of three specimens were tested. The optimal setting time of the FA/OPC and CDW/OPC binders were determined according to the procedure described in ASTM C191 [35] (method B). The evolution of the total heat of hydration (48 h) were evaluated by means of an I-Cal 8000 isothermal calorimeter (Calmetrix, Boston, MA, USA). For the calorimetric analysis of the FA/OPC and CDW/OPC binders, a comparison was made with a 100% OPC-based paste (GP type) and a paste based on the 100% OPC.

### 2.3. Mortars, Concretes, and Blocks: Production and Characterization

In order to classify the FA/OPC and CDW/OPC hybrid systems in accordance with the provisions of ASTM C1157 [36] and NSR 10 [37], the compressive strength was determined at 7, 28, and 90 days (≈25 °C) of standard mortars produced following the procedure described in ASTM C305 [38]. The test specimens correspond to cubes with a side of 50.8 mm (ASTM C109, [39]). The fine aggregate used for the manufacture of the mortars was FRA; the mortars were manufactured in cement/FRA ratios (C:A) 1:1, 1:2, and 1:2.75 to evaluate the effect of FRA on the compressive strength of the mortars. Subsequently, concretes of the two hybrid systems (HAAFA and HAACDW) were elaborated, based on the optimal proportions shown in Table 3. The ratio (L/S) used was 0.37. It should be clarified that L represents the water content present in the mixture and S includes the solid phase represented by the precursors and the activator (Na_2_SO_4_). Curing was carried out at room temperature and relative humidity greater than 90%. In the concrete, the settlement was determined in the fresh state, and in the hardened state, the compressive strength was evaluated at ages of 7, 28, 90, 180, and 360 days of curing (ASTM C39, [40]), splitting tensile strength at the age of 28 days (ASTM C496 [41]), and absorption, porosity (ASTM C642 [42]), and suction capillary at ages 28 and 180 days (ASTM C1585, [43]). For each test, a minimum of three specimens by age were used. Additionally, an electron microscopy (SEM) study was carried out to determine the state of the paste–aggregate interface in each type of concrete.

Finally, perforated and solid blocks, and concrete paving stones were fabricated, which were physically–mechanically characterized according to ASTM C140 [44], NTC 4026 [45] and NTC 2017 [46] standards. In all cases, the data reported in the physical and mechanical tests correspond to the average of three specimens.

## 3. Results and Discussion

### 3.1. Characterization of Hybrid Cement (Binder) Based on FA and CDW

**Figure 2:**
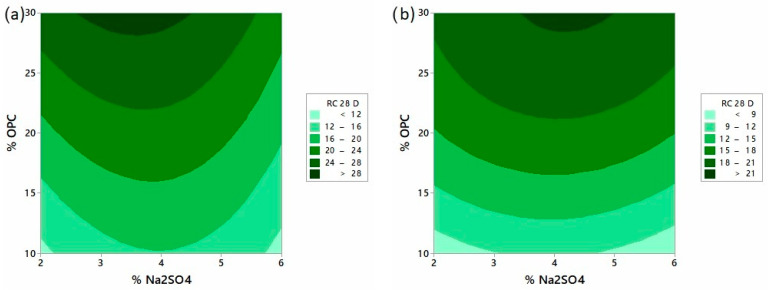
Compressive strength at 28 days of curing: effect of Na_2_SO_4_ and OPC content (wt.%). (**a**) FA/OPC, (**b**) CDW/OPC hybrid systems (pastes).

The effect of the % Na_2_SO_4_ and % OPC ratios on the compressive strength of the FA and CDW systems can be observed in the contour diagrams presented in Figure 2. The results after 28 days of curing indicate that, in the FA/OPC and CDW/OPC systems, regardless of the OPC content, there is an optimal percentage of Na_2_SO_4_, which is approximately 4%, agreeing with other reports [29,47,48]. On the contrary, upon increasing the proportion of OPC in the mixture, a significant effect on compressive strength is observed, coinciding with what was reported in [27,49]. From these results, the FA/OPC and CDW/OPC systems with 30% OPC and activated with 4% Na_2_SO_4_ (by mass) were selected, values that returned compressive strengths of 28 MPa and 21 MPa at 28 days of curing.

**Figure 3:**
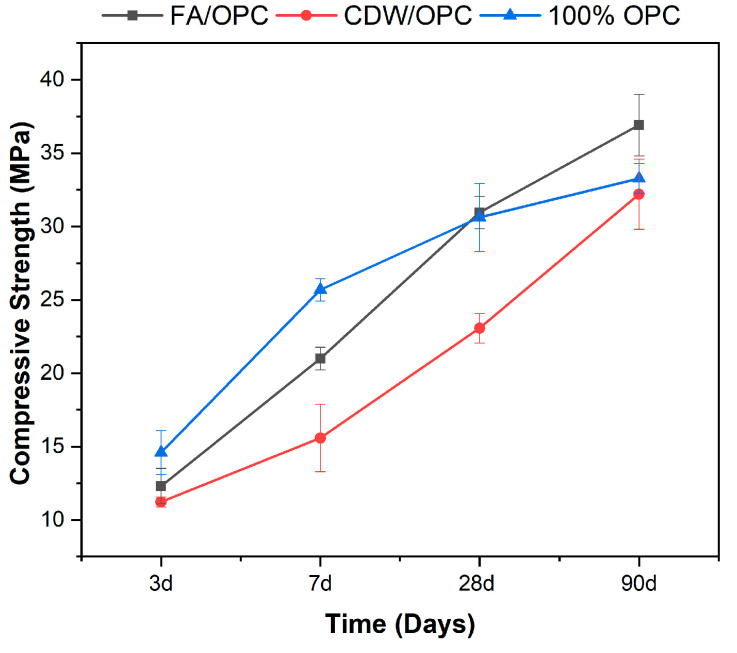
Evolution of compressive strength of hybrid cements based on FA and CDW (optimum mix): comparison with a 100% OPC-based paste (reference mix).

Figure 3 represents the evolution of the compressive strength of the optimal hybrid systems FV/30OPC and CDW/30OPC as a function of curing time (1–90 days) compared to a 100% OPC reference paste. In general, a tendency to increased resistance is observed with the evolution of the curing time, and the superior performance of the reference mixture stands out. However, it is evident that between 28–90 days of curing, FA/30OPC presents a greater strength gain, reaching 37 MPa after 90 days, a value 13% higher than that reported for the reference paste (100% OPC). This behavior agrees with what was reported by [22,27,48,49], who obtained similar strength increases for systems of alkaline activation activated with sodium sulphate at long ages, which can be attributed to the greater formation of gels of type (N,C)-A-S-H, C-A-S-H, carbonates, and ettringite as the curing time increases, generating a greater densification of the matrix [50,51].

Table 4 shows the results obtained using calorimetric analysis and the initial and final setting times (ASTM C191, [35]) of FA/OPC and CDW/OPC pastes. The greater heat released in the FA/OPC hybrid systems (94.03 J/g) may be related to the greater FA reactivity, which coincides with the compressive strength results of these systems, compared to the CDW/OPC hybrid systems. In this system, shorter setting times were also obtained.

**Figure 4:**
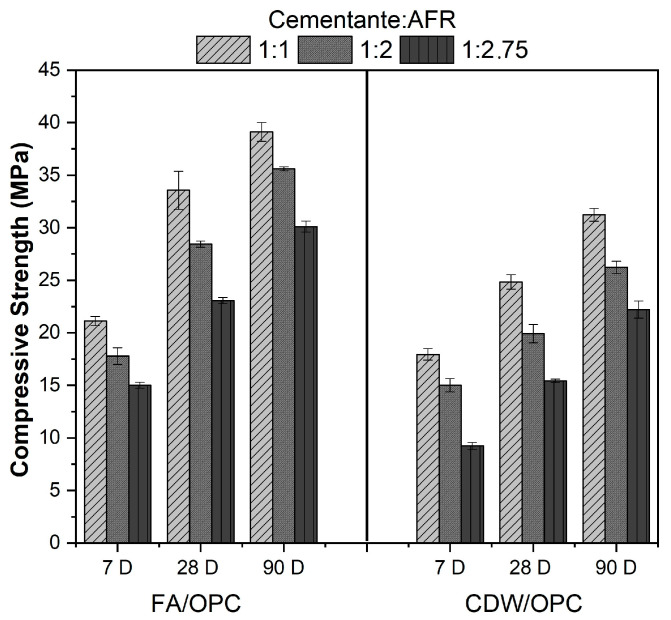
Evolution of the compressive strength of hybrid cement mortars based on FA and CDW, using FRA.

The effect of the proportion of sand in the mortars produced with these pastes is presented in Figure 4. In general, an increase in the proportion of FRA negatively affects the strength performance, regardless of the system and curing age. Indeed, the compressive strength of the FA/30OPC mortar in a 1:1 binder:FRA ratio attained 32.27 MPa after 28 days, compared to 28.44 and 23.06 MPa values reported for the 1:2 and 1:2.75 ratio mortars, respectively. The CDW/30OPC mortars in a 1:1 binder:FRA ratio report strength of 25 MPa at 28 days. According to NSR-10 Chapter D.3 (Quality of materials in structural masonry) [37], the glue mortars used in masonry constructions must meet certain levels of consistency and mechanical strength. In relation to the above, the FA/30OPC mortars activated with 4% Na_2_SO_4_ (by mass) with binder:FRA ratios of 1:1, 1:2, and 1:2.75 meet the mechanical specifications to be classified as type H mortars (minimum resistance of 22.5 MPa). CDW/30OPC mortars activated with 4% Na_2_SO_4_ (by mass) with a binder:FRA ratio of 1:1 also classify as type H adhesive mortar; while the cementitious:FRA 1:2 ratio, which reported a compressive strength value of 20 MPa, is classified as type M glue mortar; and the binder:FRA 1:2.75 ratio as type S glue mortar.

Of the two alkaline activated systems, the best strength performance corresponds to FA/30OPC, which could be related to the smaller particle size of FA, as well as a higher reactivity, coinciding with what was reported by [49,52]. The mortars corresponding to the CDW/OPC hybrid system, although they fail to surpass the compressive strength of the FA/OPC mixtures in any of the proportions, after 90 days of curing attain strengths of 31.22 MPa (binder:FRA 1:1), 26.34 MPa (binder:FRA 1:2), and 22.45 MPa (binder:FRA 1:2.75).

### 3.2. Characterization of Hybrid Concrete Based on FA (HAAFA) and CDW (HAACDW)

**Figure 5:**
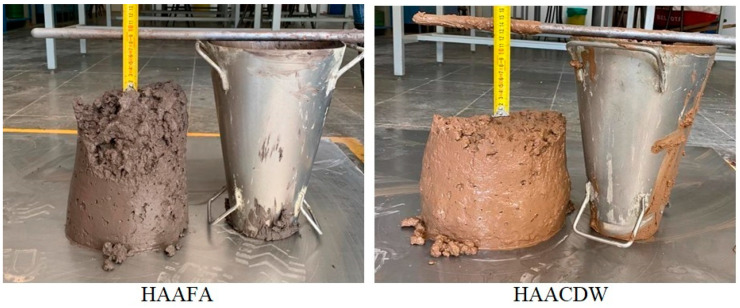
Concrete slump-flow test.

Figure 5 shows the results of the settlement test in the concrete produced with the cementitious FA/30OPC and CDSW/OPC using CRA and FRA and activated with Na2SO4; these were 90 mm for the HAAFA concrete and 140 mm for the HAAFA concrete from HAACDW. In general, the two mixtures are cohesive and do not present segregation and bleeding phenomena.

**Figure 6:**
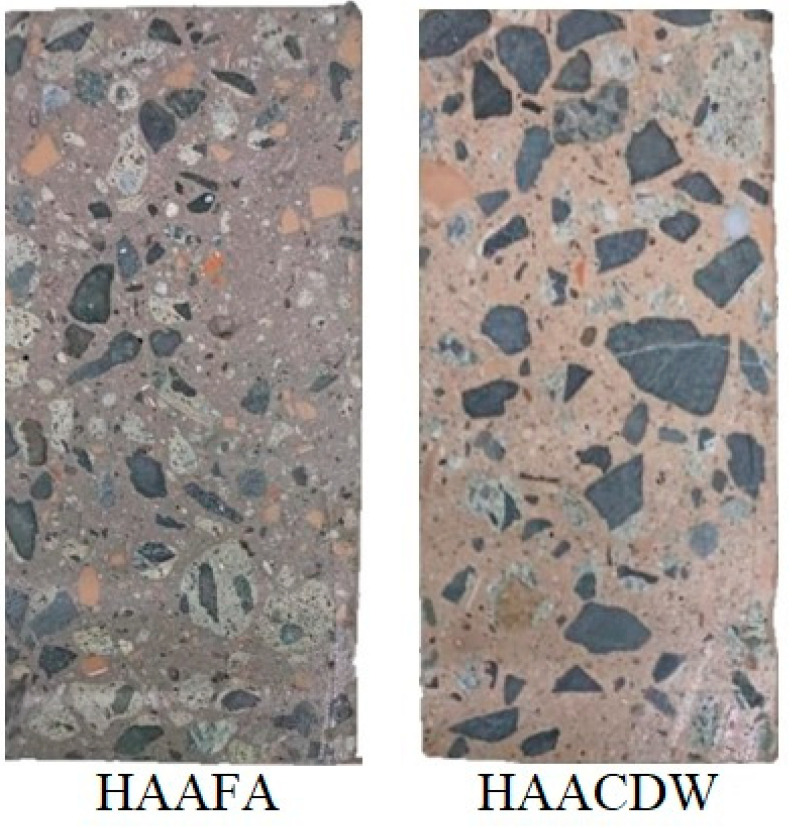
Alkaline-activated concrete in hardened state (cross section of 3 × 6 inch cylindrical specimens).

In hardened state, the concretes presented adequate superficial appearance and perfect distribution of the aggregates. The color of the concretes differs based on the raw materials used: in the first case it is due to the dark gray tone of FA, while in the second it is attributed to ceramic waste (red) and masonry waste (red); these shades can be an advantage at the application or industrial level (Figure 6).

**Figure 7:**
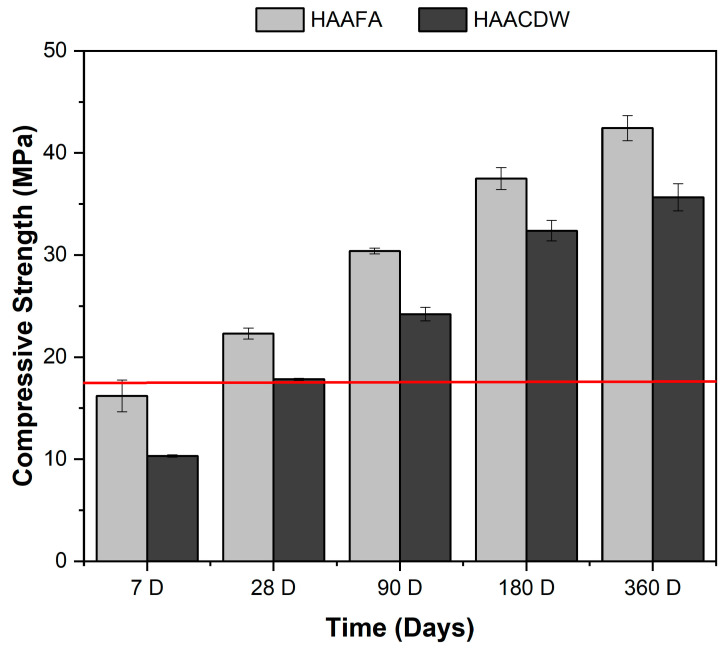
Compressive strength of concrete as a function of curing times.

The results of the compressive strength at the ages of 7, 28, 90, 180, and 360 days of curing are presented in Figure 7. For the two hybrid concretes evaluated, there is an increase in the compressive strength with the curing time, a characteristic behavior of Portland cement-based concrete. Likewise, a marked difference can be seen between the different alkaline activation systems.

The lowest compressive strength was identified in hybrid concretes based on CDW. These materials report a RC of 10.32 MPa at 7 days of curing, exhibiting a 73% increase in strength at a curing age of 28 days. At 90 days of curing, it is possible to obtain strength of up to 24.21 MPa. This is associated with the low reactivity that CDW might have, which causes the greatest increases in strength to be obtained at prolonged ages of curing. This coincides with what was reported by Robayo-Salazar et al. [53], who affirmed that this behavior is related to the slow reactivity presented by CDW. For the FA-based hybrid systems, higher RC values are observed at the different ages of curing. The HAAFA concrete attained a strength value approximately 1.6 times higher than the HAACDW concrete after 7 days of curing. Indeed, this mixture (HAAFA) reported a compressive strength of 16.19 MPa after only 7 days of curing, showing an increase of 88% at 90 days (30.39 MPa). It should be noted that after 28 days of curing, the HAAFA and HAACDW concretes attained 22 and 18 MPa, respectively, exceeding the limit of 17 MPa established as the minimum RC to be considered structural concrete according to Title C of NSR-10 (red line in Figure 7). The greatest increase in the mechanical performance of hybrid concretes (HAAFA) is related to the greater reactivity of FA compared to CDW [30,33]. In general, in both systems, the presence of hybrid gels (N,C-A-S-H) and ettringite contributes significantly to the compressive strength of the final products, and in turn decreases porosity by densifying the material [54,55]. At curing ages of 180 and 360 days, it stands out that the HAACDW hybrid concretes are those with a greater increase in strength compared to the curing ages of 90 days. They manage to increase by 47%, while in the HAAFA an increase of 40% is achieved; it is noteworthy that the HAAFA and HAACDW concretes reach 360-day strengths of 42 MPa and 36 MPa, respectively. Based on these results, it can be said that the activation with Na_2_SO_4_ of hybrid systems based on FA and CDW are effective and contribute significantly to the increase in the mechanical strength of concrete.

**Figure 8:**
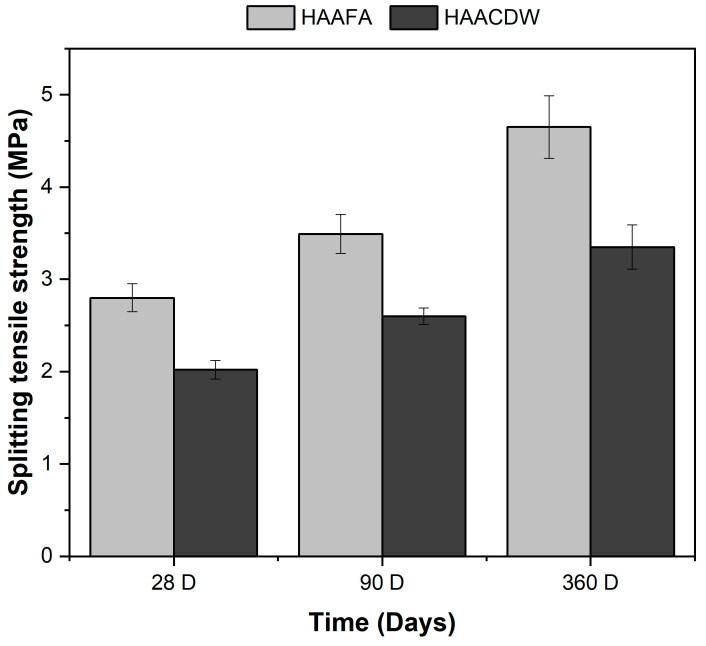
Indirect tensile strength of concrete at 28, 90, and 360 days of curing.

The results of indirect tensile strength of hybrid concretes activated with Na_2_SO_4_ and cured at room temperature are shown in Figure 8. The trend is similar to that obtained for compressive strength. After 28 days of curing, the behavior of the HAAFA concretes stands out, which present an indirect tensile strength of 2.08 MPa, exceeding the HAACDW concrete by 38%. At 90 days of curing the HAAFA and HAACDW concretes, the tensile strength increases by 25% and 22%, respectively, with respect to the samples evaluated at 28 days [56]. At the final ages of the trial (360 days), the behavior of the HAAFA stands out, which presented an increase of 39% with respect to the specific HAACDW.

**Figure 9:**
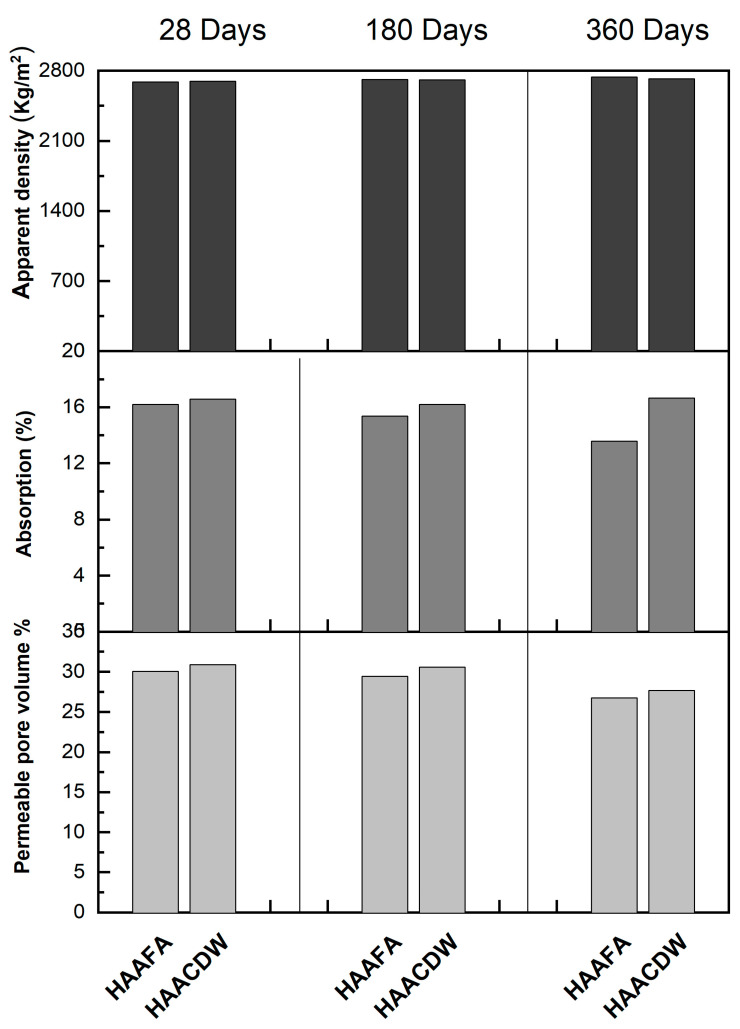
Density, absorption, and porosity of HAAFA and HAACDW concrete at 28, 180, and 360 days of curing.

The density, absorption percentage and permeable pores of the concrete under study are presented in Figure 9. It is identified that the apparent density values obtained for these materials are close to those reported in the literature for conventional Portland cement-based concrete (with natural siliceous-type aggregates), which have a density value between 2300 to 2600 kg/m^3^ [57]. There are no significant differences in the density of the material evaluated at 28 days of curing with respect to that reported at advanced ages of curing (180 and 360 days); this result contrasts with the one obtained in previous studies [56] for concretes based on alkali-activated GBFS (100%) where a significant increase in the density of these materials was observed with the advance of curing age. It is observed that the degree of reaction of each one of the concretes, as expected, increased with the curing time, which contributed to decrease the total absorption and the porosity of the concretes. However, it is highlighted that throughout the curing time the HAAFA concretes presented lower percentages of absorption and permeable pores, which is directly associated with the higher RC previously reported.

Upon comparing the absorption percentages of the alkaline activation concretes, the HAAFA samples stand out, which at 28, 180, and 360 days of curing present the lowest percentages (16.21, 15.37%, and 13.58%, respectively) compared to the HAACDW concretes. The volume of permeable pores presents a similar tendency to absorption in the two concretes at different ages of curing (Figure 9). It should be noted that the high values of absorption and permeable pores may be related to the recycled aggregates used (CRA and FRA), which have greater absorption.

**Figure 10:**
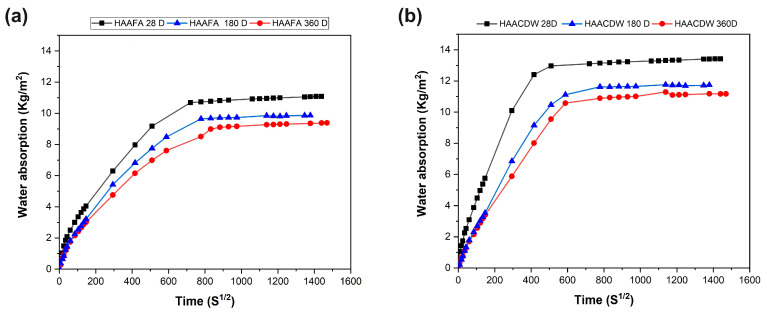
Capillary suction curves of hybrid concrete at different curing ages. (**a**) HAAFA and (**b**) HAACDW.

The capillary suction curves obtained for the concrete at the different curing ages are shown in Figure 10. It can be seen that at higher curing ages the samples present significantly lower water absorption, which is consistent with lower porosity and a refined pore structure. In general, the lower capillary water absorption for concrete at higher curing ages is consistent with the results obtained in mechanical strength and total porosity. The HAACDW concrete (Figure 10b) at a curing age of 28 days is the system with the highest water absorption, compared to the HAAFA samples (Figure 10a).

**Figure 11:**
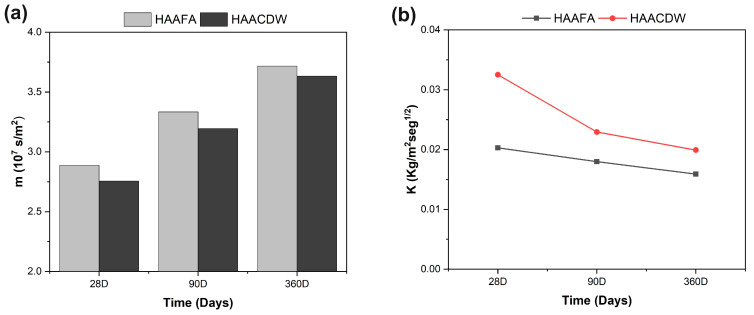
(**a**) Resistance to water penetration “m”, and (**b**) coefficient of capillary absorption of HAAFA and HAACDW hybrid concretes with 28, 180, and 360 days of curing.

Figure 11 shows the behavior of the resistance to water penetration (m) and the capillary absorption coefficient (K) of the concretes. It is observed that at advanced ages of curing there is a reduction in “K” of up to 63%, compared to what was reported at 28 days of curing, where the lowest values correspond to the HAAFA concrete (Figure 11b). This behavior coincides with what was reported in [58]. In turn, the resistance to water penetration “m” tends to increase at higher curing ages; it should be noted that the two concretes show very similar values of “m” (Figure 11a). The decrease in the capillary absorption coefficient and the increase in the resistance to water penetration are related to the greater densification of the concretes due to the increase in the reaction products formed and consequently the refinement of the pores in the matrix [58,59,60].

**Figure 12:**
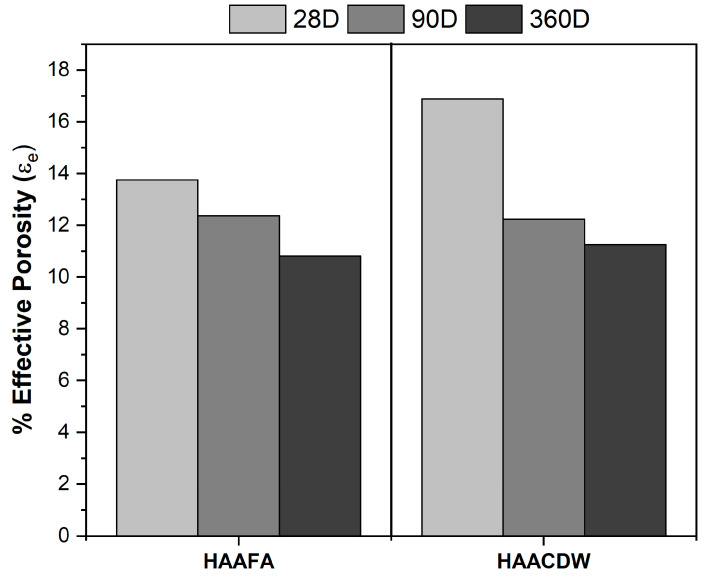
Effective porosity of HAAFA and HAACDW concretes with 28, 180, and 360 days of curing.

Figure 12 presents the effective porosity of the concrete at the different curing ages. Generally, the decrease in this property can be seen with the increase in curing time because of the progress of the reactions. After 28 days of curing, HAAFA presents an effective porosity of 13.7%, while in the HAACDW concretes it was 16.8%; at 360 days, approximately similar porosities are achieved (11%).

**Figure 13:**
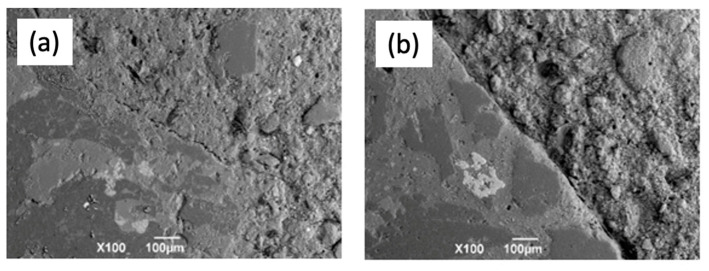
SEM of the boundary between the aggregate and the concrete matrix. (**a**) HAAFA and (**b**) HAACDW.

Figure 13 shows the microphotographs obtained using scanning electron microscopy after 28 days of curing. In the HAAFA concrete (Figure 12a), a dense matrix with some microcracks is observed, the interfacial transition zone between the aggregate and the paste presents a good union; in some zones, cracks or spacing between the recycled aggregate and the matrix were observed. However, good interaction between the aggregate and matrix is appreciated, coinciding with the good mechanical performance reported from the samples. On the contrary, in the HAACDW concrete, a porous matrix is observed, and the interfacial transition zone between the recycled aggregate and the matrix does not present a good interaction, which causes a spacing between the aggregate and the matrix (Figure 12b), which may be the result of the lower compressive strength and higher reported porosity.

### 3.3. Production and Characterization the Construction Elements

Construction elements such as hollow blocks, solid blocks, and paving stones were produced from HAAFA and HAACDW. Table 5 includes the technical specifications according to the NTC 4026 standard [45], based on which the results of the physical–mechanical characterization of the blocks was analyzed, which is presented in Table 6.

In accordance with NTC 4026, entitled “Concrete units (blocks and bricks) for structural masonry”, three classes of concrete masonry units are established: normal weight (2000 kg/m^3^ or more), medium weight (between 1680 and 2000 kg/m^3^), and light weight (<1680 kg/m^3^). Regarding weight, the HAAFA and HAACDW drilled blocks reported a density of 1846kg/m^3^ and 1880 kg/m^3^, respectively, being classified as medium-weight blocks. The compressive strength at 28 days of curing for the HAAFA and HAACDW perforated blocks was 15.28 MPa and 9.6 MPa, respectively, and according to NTC 4026, the blocks made with HAAFA can be classified as high-class structural units, and the block made with HAACDW as a low-class structural unit. According to the maximum limits of water absorption defined in NTC 4026, the two elements comply with what is specified in the standard. The solid blocks are also considered (Table 6) of medium weight, and the strength far exceed the regulations to be classified as high class; however, the one produced with HAACDW presents an absorption value higher than that specified, for which is considered low class.

In the case of the concrete paving stones, Table 7 presents the results of the physical-mechanical characterization according to the requirements established by the NTC 2017 standard [46], entitled “Concrete paving stones for pavements”. According to this standard, concrete paving stones that meet the physical–mechanical requirements are suitable for building pavements for pedestrian traffic, vehicular traffic (including port yards and cargo terminals, airports, transportation terminals, service stations, warehouses, etc.), and distributed static loads (bulk storage warehouses). The classification of the paving stone is given in the NTC 2017 standard, according to its geometry or shape, physical requirements and mechanical resistance. Regarding the geometry of the paving stones produced in the present study, it corresponds to a type 2 “dog bone” paving stone with an “I” shape. Regarding water absorption, the NTC 2017 establishes that concrete paving stones must have a total water absorption of no more than 7% as an average value and modulus of rupture (MOR) between 4.2–5.0 at 28 days of curing.

As such, the HAAFA and HAACDW paving stones reported a modulus of rupture of 5.3 MPa and 4.6 MPa, respectively, values that are within the acceptance range. However, the percentage of water absorption of both paving stones (9.5% (HAAFA) and 10.8% (HAACDW)) exceeds the maximum established by the NTC 2017. It should be noted that the level of water absorption of the prefabricated units that were produced is influenced by the placement and compaction process that was carried out, being semi-automatic in this case.

Additionally, using a percentage of 100% recycled aggregates in the mixes promotes greater water absorption due to the greater porosity of this type of aggregate. Based on this, it is expected that if the percentage of FRA and CRA is reduced, at the same time that the production system is more industrialized or the level of compaction and vibration increases during the forming process of these elements, the levels of absorption are significantly reduced.

## 4. Conclusions

The results of this research demonstrate the possibility of producing alkaline-activated hybrid materials (cements, mortars, concretes, and precast elements) based on fly ash and construction and demolition waste activated with sodium sulfate and recycled aggregates as a sustainable alternative to conventional OPC-based materials. The following conclusions can be drawn from the experimental results:

Hybrid cements produced from FA and CDW activated with sodium sulfate and 30% OPC reported strengths of 37 MPa and 32 MPa, respectively, and the corresponding mortars including recycled fine aggregate meet the specifications for adhesive mortars recommended in the NSR 10 Title D.

The hybrid alkaline activation concretes activated with sodium sulfate (HAAFA and HAACDW) and using CDW as aggregates, meet the minimum resistance (17 MPa) of the NSR-10 title C for their classification as structural concretes. At curing ages of 360 days, it is highlighted that the HAAFA concrete achieves strength of approximately 42 MPa and the HAACDW concrete of 36 MPa.

The production and physical–mechanical characterization of the perforated blocks, solid blocks, and paving stones obtained from the HAAFA and HAACDW concretes demonstrated the potential application for precast manufacture.

## Figures and Tables

**Table 1:** Chemical composition of raw materials.

Material	SiO_2_	Al_2_O_3_	Fe_2_O_3_	CaO	MgO	Na_2_O	SO_3_	TiO_2_	Others	LOI ^1^
FA	59.03	23.97	5.98	0.74	0.31	0.19	0.55	0.95	1.93	6.35
CDW	47.60	11.21	5.94	21.21	1.19	0.67	-	-	2.32	9.86
OPC	19.13	4.42	4.32	57.70	1.60	-	2.32	-	0.73	9.78

^1^ LOI: loss on ignition.

**Table 2:** Characteristics of the recycled aggregates (FRA and CRA) obtained from CDW.

Characteristics	Fine Recycled Aggregate (FRA)		Coarse Recycled Aggregate (CRA)	
	Standard	Experimental Value	Standard	Experimental Value
Bulk density (kg/m^3^)	ASTM C128 [29]	2029	ASTM C127 [30]	2326
Absorption (%)	ASTM C128 [29]	12.12	ASTM C127 [30]	9.17
Unit weight (kg/m^3^)	ASTM C29 [31]	1240	ASTM C29 [31]	1211
Maximum size (mm)	N/A		ASTM C136 [32]	25.4
Fineness modulus	ASTM C136 [32]	2.63	N/A	
Organic impurities	ASTM C40 [33]	Organic plate No. 1	N/A	
Resistance to degradation (%)	N/A		ASTM C131 [34]	33.65

**Table 3:** Design of concrete mixes.

Material	HAAFA (kg)	HAACDW (kg)
Precursor FA	350	--
Precursor CDW	--	350
OPC	150	150
Sodium sulphate (Na_2_SO_4_)	20	20
Water	182	182
Coarse recycled aggregate (CRA)	670	695
Fine recycled aggregate (FRA)	670	695
Total	2040	2092

**Table 4:** Setting time and heat of reaction.

Sample	Initial Setting Time (Min)	Final Setting Time (Min)	Heat of Reaction (J/g)
FA/OPC	88	146	94.03
CDW/OPC	350	570	80.13

**Table 5:** Technical specifications for the structural blocks according to NTC 4026 [45].

Compressive Strength at 28 Days (Rc 28), Evaluated on the Average Net Area (Anp) (Minimum, MPa)			Water Absorption (Wa) (Maximum %) Based on Weight (Density) (kg/m^3^)		
Class	Average of 3 Units	Individual	Light Weight, Less than 1680 kg/m^3^	Medium Weight, 1680 kg/m^3^ to Less than 2000 kg/m^3^	Normal Weight, 2000 kg/m^3^ or More
High	13	11	15	12	9
Low	8	7	18	15	12

**Table 6:** Properties and characteristics of the perforated and solid block manufactured from the hybrid concretes HAAFA and HAACDW.

Type of Block	Rc (28d) MPa	Weight (kg/m^3^)	Wa %
HAAFA perforated block (high class)	15.28 ± 0.5	1846	11.4
HAACDW perforated block (low class)	9.6 ± 0.6	1880	13.6
HAAFA solid block (high class)	29.3 ± 1.3	1874	11.8
HAACDW solid block (low class)	22.5 ± 0.8	1790	14.1

**Table 7:** Properties and characteristics of paving stone made from HAAFA and HAACDW hybrid concrete.

Paving Stone	Module of Rupture (MOR) at 28 Days (Minimum, MPa)		Water Absorption (Wa) (% Maximum)	
	NTC 2017 [46]	Experimental Result	NTC 2017 [46]	Experimental Result
HAAFA	4.2–5.0	5.3 ± 0.3	7	9.5
HAACDW		4.6 ± 0.5		10.8

## Data Availability

Data is contained within the article.
